# Fiber-reinforced scaffolds in soft tissue engineering

**DOI:** 10.1093/rb/rbx021

**Published:** 2017-08-04

**Authors:** Baoqing Pei, Wei Wang, Yubo Fan, Xiumei Wang, Fumio Watari, Xiaoming Li

**Affiliations:** 1 Key Laboratory for Biomechanics and Mechanobiology of Ministry of Education, School of Biological Science and Medical Engineering, Beihang University, Beijing 100191, China; 2 State Key Laboratory of New Ceramic and Fine Processing, Tsinghua University, Beijing 100084, China; 3 Department of Biomedical Materials and Engineering, Graduate School of Dental Medicine, Hokkaido University, Sapporo 060-8586, Japan

**Keywords:** scaffolds, reinforce, fibers, soft tissue

## Abstract

Soft tissue engineering has been developed as a new strategy for repairing damaged or diseased soft tissues and organs to overcome the limitations of current therapies. Since most of soft tissues in the human body are usually supported by collagen fibers to form a three-dimensional microstructure, fiber-reinforced scaffolds have the advantage to mimic the structure, mechanical and biological environment of natural soft tissues, which benefits for their regeneration and remodeling. This article reviews and discusses the latest research advances on design and manufacture of novel fiber-reinforced scaffolds for soft tissue repair and how fiber addition affects their structural characteristics, mechanical strength and biological activities *in vitro* and *in vivo.* In general, the concept of fiber-reinforced scaffolds with adjustable microstructures, mechanical properties and degradation rates can provide an effective platform and promising method for developing satisfactory biomechanically functional implantations for soft tissue engineering or regenerative medicine.

## Introduction

Soft tissue as a broad term covers a variety of tissues such as fat, skin, tendon, muscle, articular cartilage, nerves, fascia, intervertebral disc, synovium, joint capsule and blood vessels [1], and these tissues usually have the functions of surrounding, supporting or connecting the body structure and organs. As a frequent occurrence in everyday life, soft tissue damage caused by congenital defects, disease, trauma and aging often leads to non-self-healable contour malformations [2]. So far, autologous implantation is the primary method of treating these defects or diseases, however, its main drawback is that autologous tissue can be easily absorbed and rapidly lost in volume, so only 40–60% of soft tissue cells remain viable [3–5]. In addition, donor site allogeneic response and morbidity also limit the widespread use of autologous transplantation [6]. As a relatively new and attractive approach, tissue engineering has been used to develop new biological substitutes to overcome barriers of current clinical treatments for repairing and regenerating damaged or diseased soft tissues and organs [7].

Tissue engineering can be simply defined as preparing a living tissue construct by expanding cells *in vitro* and incorporating the cells into a temporary scaffold to mimic the structure and function of the native tissue. The scaffold as artificial extracellular matrix (ECM) with specific appearances and functions plays a pivotal role in promoting cell growth and differentiation, following the growth patterns and rules found in natural tissues and organs [8]. ECM in native tissue is a three-dimensional (3D) network whose composition and structure can interact with cells continuously to provide structural support, transfer mechanical forces and transmit chemical signals in native tissues. In general, scaffolds for tissue engineering should possess sufficient mechanical properties, biocompatibility and appropriate morphology to support cell inward growth, and have high porosity and interconnection to transmit regulatory chemical signals, nutrients, oxygen and metabolic wastes [9–11].

In the past few years, various biomaterials have been used to prepare scaffolds in soft tissue engineering, including natural materials, such as collagen, gelatin and elastin [12, 13] and synthetic materials, such as poly(ɛ-caprolactone)(PCL), poly(glycolic acid)(PGA), poly(lactic acid)(PLA), poly(hydroxy alkenoates) (PHAs) and their copolymers [14, 15]. However, many biomaterials are difficult to meet the mechanical properties of engineering scaffolds due to their own limitations [16]. Recent research results have shown that mechanical properties, such as elasticity, stiffness and strength, are essential factors that directly influence the ability of cell adhesion, proliferation and differentiation [17–19]. When soft tissues have high mechanical activity, such as dermis, blood vessels and heart valves, the scaffold has sufficient mechanical properties that are particularly important to the effective transfer of mechanical stimuli [17, 20, 21]. For example, the failure of the scaffold grafted in the blood vessel is usually due to intimal hyperplasia caused by the compliance mismatch between the graft and the host [22]. In addition, bladder tissue studies have also shown that artificial membranes with elastic modulus closer to natural bladder tissue can promote cell proliferation [23]. In fact, the mechanical properties of the scaffolds should maintain at a sufficient level during the tissue growth period. However, current scaffolds used to repair soft tissue defects often cannot meet the balance of biomechanics and tissue regeneration performance.

The native ECM of biological soft tissues is usually a fiber network with complex constituent and microstructure. The main components of soft tissue are elastin and collagen fibers [24, 25]. Elastic proteins have the most linear elasticity, while collagen fibers are the main load elements that have a direct effect on the physical and mechanical properties of soft tissue. Therefore, many soft tissues, such as blood vessels [26], heart valve leaflets [27] and cardiac muscle [28], are composed of collagen fibers embedded in a gel-phase matrix of elastin, and their arrangement and microstructure combine to determine the biomechanical properties of soft tissue. For example, articular cartilage is made up of stiff and strong collagen fibers embedded in a proteoglycans matrix [29]. According to the current research of soft tissues, collagen fibers determine the stiffness and strength in tension and alleviate the compression-dependent response in compression, while the hydrogel-like elastin establishes a fluid pressure mechanism to support and distribute the compressive load [30, 31].

As mentioned above, a number of damaged tissues or organs have fiber-reinforced hydrogel constructs, and thus the concept of fiber-reinforced scaffolds is a relatively new but effective measure for more accurately remodeling the structure and function of native tissue. Since fiber-reinforced scaffolds have the potential to improve the performance of the material, they have been used to enhance soft tissue repair scaffolds, such carbon fiber-, natural fiber-, glass fiber- and synthetic fiber- reinforced scaffolds [32–34]. Recent studies have shown that fiber-reinforced scaffolds can provide adequate mechanical environment during the healing state and improve biocompatibility and, in most cases, biodegradation of scaffolds, which facilitates cell adhesion, migration, differentiation and proliferation. Furthermore, the mechanical properties of fiber-reinforced scaffolds are affected by fiber orientation, aspect ratio and volume fraction, matrix properties and the binding of the matrix and fibers, thus the mechanical properties of fiber-reinforced scaffolds could be controlled by adjusting these factors [1, 35–38]. Fiber-based materials or structures have been widely used as reinforcing additives in tissue engineering and provide a promising optimization method for the development of implant scaffolds for soft tissue engineering. The purpose of the review is to provide an overview of the current research and development of new fiber-reinforced scaffolds for soft tissue repair.

## Fiber-reinforced scaffolds for cartilage tissue repair

Cartilage tissue often gets hurt in daily life and cannot be completely self-healing, especially in the elderly, because the matrix turnover, vasculature and mature chondrocytes ability of cartilage are relatively weak [1, 39]. Cartilage tissue engineering has become a promising treatment to cover the shortage of current surgical and nonsurgical treatment, such as autograft, allograft and micro fracturing, which are linked with dissatisfaction, inefficiency and low success rates [40]. The scaffolds for cartilage engineering have recently received considerable attention in academia and industry because of their potential benefits, such as cost-effective, time-efficient, and single procedure, non-donor site morbidity and the possibility of cell therapies. At present, a majority of the popular materials have been used to prepare cartilage scaffolds, but most of the products fail to meet the critical requirements of sufficient mechanical strength and structural resilience.

**Figure 1.**
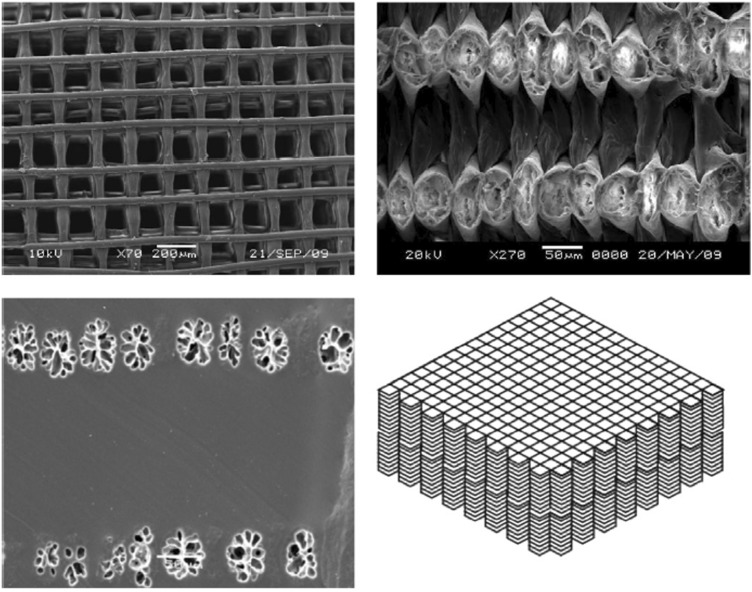
(**A**) SEM Image of polyurethane construct (scale = 100 lm). (**B**) Cross-section image of the 200 × 200 construct (scale = 50 lm). (**C**) Cross-section image of reinforced composite gel; the pores correspond to the fiber and the smooth surface corresponds to the gel (scale = 50 lm). (**D**) Sketch of the processing method. (Adapted with permission from Ref. [41]. Copyright 2013 Elsevier Ltd)

Among many studies, impressive results have been achieved on the use of fibers to improve or enhance the mechanical properties of cartilage scaffolds. For example, Agrawal *et al.* [41] designed a new type of elastomeric fiber-reinforced hydrogel composite to achieve a cartilage-like structure by impregnating elastic polyurethanes fibers, that had been arranged into crossed ‘log-piles’, with an epoxy-amine hydrogel from polyethyleneglycol diglycidylether (Fig. 1). They found that when the fibers were parallel to the stress axis, the elastic modulus of the reinforced composites at 3.68 ± 0.24 MPa had a two-fold increase as compared to the epoxy-amine hydrogel at 1.74 ± 0.03 MPa. At the same time, the fracture strain at 3–12 kJ m^−^^2^ was significantly higher than that of native cartilage around 1 kJ m^−^^2^. However, it is noteworthy that the mechanical properties of the scaffold cannot be too large, that may result in high shear and high relative stress concentration of the adjacent cartilage. This work showed that the fibers could provide the strength and elasticity and enrich the shape and structure of the gel scaffold to make it closer to natural cartilage tissues. The polyurethane cannot be rapidly degraded and absorbed, which may cause adverse reactions in the body, hindering the normal growth of cells and the formation of new tissues. Unlike the polyurethane, the poly(a-hydroxy acid) family is the most common bioresorbable polymers that have the significant advantages for the preparation of scaffolds with efficient cell-seeding properties to support long-term cartilage repairing and healing *in vitro and in vivo.* Slivka *et al.* [42] prepared a resorbable PGA fiber-reinforced poly(_D, L_-lactide-co-glycolide) (PLGA 75:25) scaffold for cartilage repair by using the combined manufacturing technique of the dissolution precipitation and vacuum. The yield strength and the compressive modulus of the reinforced composite increased from 0.7–2.5 MPa and 10–50 MPa, respectively, when the weight fraction of PGA fiber increased from 0 to 20%. In addition to obtain optimized mechanical strength, the scaffold possessed improved pore size and morphology, and the fibers might guide the distribution and uniformity of the pore. The articular cartilage scaffold requires not only a mechanical environment but also a highly porous interconnected structure suitable for the proliferation and precipitation of chondrocytes. However, these two objectives may be opposed to each other, because with the increase in porosity and interconnection, the mechanical properties of the scaffold may be reduced. As reported by Slivka *et al.* some other studies also found that fiber-reinforced scaffolds for cartilage repair have the ability to improve mechanical properties while maintaining high porosity. For example, Visser *et al.* [43] used highly organized, high-porosity PCL microfiber networks to reinforce gelatin methacrylamide (GelMA) hydrogel scaffolds based on the melt-electrospinning writing technology. Compared with pure hydrogel or microfiber scaffolds, the stiffness of the reinforced scaffold (405 ± 68 kPa) was significantly increased by 54-fold, similar to that of natural articular cartilage (400–800 kPa). Their stress-strain curve and elasticity were also close to those of the native articular cartilage. And the reinforced scaffold also has a high porosity of 93–98%, which achieved the desired target of the idea scaffold. What’s more, GelMA has been proved to a biocompatible material that allows for matrix deposition when embedded in cells. Thus, chondrocytes cultured *in vitro* survived under physiological compression load and retained the round morphology, which highlighted the potential for establishing cell-culture platforms and developing engineering constructs for articular cartilage repair. Similarly, Visser and Boere [44] also demonstrated that the GelMA hydrogel scaffold reinforced by poly(hydroxymethylglycolide-co-ɛ-caprolactone) (pHMGCL)/PCL thermoplastic polymer fibers not only had enhanced resistance to repeated axial and rotational forces but also supported cell proliferation and differentiation for a focal articular cartilage defect. The chondrocytes embedded in these fiber-reinforced constructs produced cartilage-specific matrix components both *in vitro and in vivo*. Thus, the well characterized GelMA hydrogel system reinforced with the organized PCL or (pHMGCL)/PCL fiber structures is conducive to supporting cell growth and proliferation in a customizable, mechanically diversified environment and has the ability to guide the formation of the matrix.

In addition to the above synthetic materials, natural fibers are also studied as reinforcements to improve the performance of the hydrogel scaffold for repairing and remolding cartilage tissue. For example, Yodmuang *et al.* [45] reported one of the first fiber-reinforced hydrogel scaffolds, whose matrix and reinforcements were composed of the same material, silk from Bombyx mori. Natural silk has superior biocompatibility and degradability, and it rarely causes an immune response and can be conjugated to RGD peptide modifications or functional groups to enhance cell adhesion and proliferation. This study demonstrated that the silk-based reinforced hydrogels showed excellent chondrocyte response and supported the deposition and localization of glycosaminoglycan and collagen around the fibers. The reinforced hydrogel facilitated the development of constructs with strong mechanical properties after culturing for 42 days. Although the reinforced composites have achieved optimal load-bearing structures similar to cartilage tissue, their main advantage is to provide a biocompatible and biodegradable cartilage scaffold materials that can used to simulate the functional natural tissues. Natural polymers used as reinforcing agents may not provide as sufficient mechanical stability as synthetic materials, and their biodegradation rates may be higher than expected. In other similar studies, Mirahmadi *et al.* [46] also used chopped silk fibers to reinforce the thermosensitive chitosan/glycerophosphate hydrogel matrix to obtain a suitable mechanical hydrogel composite with biological functions that can be used as hyaline cartilage scaffolds. The addition of silk fibroin fibers improved the mechanical properties of chitosan/glycerophosphate matrix, however, the compressive modulus of the reinforced composites (about 2.8–3.5 KPa) was lower than those of articular cartilage (about 0.5–1 MPa). The results of proteoglycan and collagen type II indicated that the scaffold supported the cartilage formation phenotype of chondrocytes. In general, the results presented in the above works indicate that both natural and synthetic fiber reinforced gel scaffolds are well suited for cartilage tissue engineering. Synthetic materials can be used as well controlled units to modulate the mechanical and structural properties on the design of scaffolds, while natural materials with excellent biocompatibility and biofunctionality offer biological signals to facilitate tissue-specific interactions and highly support for cartilage matrix deposition and formation. Furthermore, the reinforced scaffold still needs to be further optimized by controlling the amount, alignment and direction of the fibers in the host matrix in order to simulate the collagen fiber orientation in superficial and subchondral regions of the cartilage to bear multi-directional loading in the joint.

## Fiber-reinforced scaffolds for tendon and ligament repair

Both ligament and tendon play an important role in maintaining the normal movement and stability of joints in the musculoskeletal system. As common injury connective tissues, there are more than 800 000 people per year need medical care due to ligament or tendon damage [47–50]. Surgical repair is the most commonly used therapeutic method, but often leads to degenerative or frayed tissue [51, 52]. As more and more allografts and xenografts are used for tendon and ligament repair, tissue engineered scaffolds as an artificial implant have also been developed to be a new prospect for the manufacture of ligament or tendon substitutes [53]. A variety of scaffolds derived from both natural and synthetic have some success as ligament or tendon substitutes, but most of them fail to provide a lasting benefit to remain a suitable bio-environment and adequate mechanical and tribological properties of the original tissue, such as elastic modulus, toughness and ultimate strength [50, 54, 55].

Numerous groups have used fiber-reinforced materials to develop biomechanically functional tissue constructs for tendon and ligaments repair to overcome the current limitations. For example, based on the carbodiimide cross-linking method, Shepherd *et al.* [56] investigated a lyophilized structures of collagen fiber-reinforced collagen–chondroitin-6-sulfate (C6S) materials to produce a bioactive and biomechanical scaffold for ligament and tendon repair, as well as cartilage. The reinforced collagen-C6S with crosslinked fibers had a significantly enhanced average ultimate tensile strength of 1.5 MP, compared with that of the collagen-C6S matrix of 0.015 MP. However, the ultimate tensile strengths of human tendons and ligaments are in the range of 30–100 MPa. The collagen-based reinforced scaffold exhibited highly bioactive and porosity, which enabled cellular infiltration, attachment and proliferation. Collagen-based reinforced material reconstituted from type I and type II collagen as a type of natural material is a promising method for ligament and tendon repair, due to their chemical composition and physical properties like natural tissue, which can promote the production and regeneration of new ECM and native tissue. Although the addition of natural fibers has the capability of improving the mechanical properties of the matrix material, it is still far away from the requirements of repairing ligament and tendon, thereby requiring further research and development.

**Figure 2.**
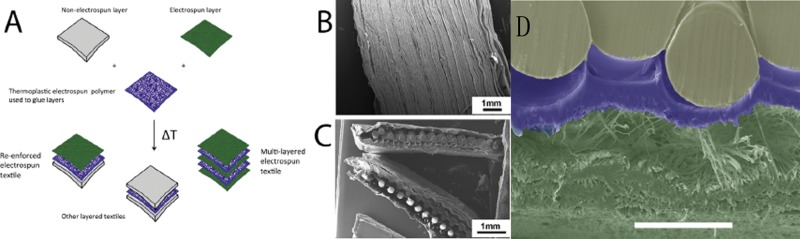
Multi-layered scaffolds from electrospun and non-electrospun mats and prototype layered electrospun/woven scaffold architecture. (**A**) The technique of thermoplastic mats and the incorporation of electrospinning and non-electrospinning. (**B**) Multi-layered electrospun sheets. (**C**) Woven polydioxanone textile sandwiched between two electrospun mats. (**D**) Schematic diagram of the prototype scaffold designed and tested. Unless specified scale bars are 100 lm. (Adapted with permission from Ref. [57]. Copyright 2015 Elsevier Ltd)

**Figure 3.**
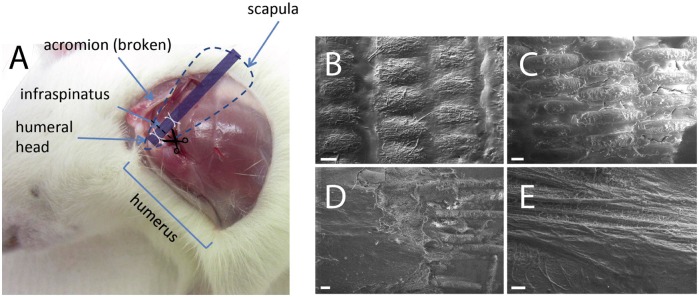
The layered scaffold integrated in an *in vivo* rat model. (**A**) An illustration of the scaffold placement in the rat shoulder. (**B**–**E**) Scanning electron micrographs show tissue ingrowth into the layered scaffold after 2 weeks (B), 4 weeks (C), 6 weeks (D) and 12 weeks (E). (Adapted with permission from Ref. [57]. Copyright 2015 Elsevier Ltd)

As for synthetic materials with adequate mechanical properties, they have a better performance in creating the suitable mechanical environment for ligament and tendon repair. For example, Santis *et al.* [25] designed a continuous fiber-reinforced polymer scaffold that was HydroThane^TM^ (Hydrophilic Thermoplastic Polyurethane by Cardio-Tech International) matrix reinforced by PET (polyethylenterephtalate) fibers to obtain the necessary mechanical properties as a substitute for tendons and ligaments. The composites reproduced typical stress-strain curves similar to those of natural tendon and ligament. This work found that the mechanical behavior of the composite could be seen as a function of the winding angle, so that the value of the elastic modulus can be easily controlled by hanging the winding angle of the fibers. At the same time, the wider alignment of the PET fibers could result in an increase in strength and modulus along the load direction. Thus, the additive fibers with the different winding angle and distribution can have a certain regular impact on the mechanical properties of the scaffold. What’s more, some related studies have reported that fiber-reinforced scaffolds made from synthetic materials have good properties in the *in vivo* implantation experiments. For example, Hakimi *et al.* [57] created a layered fiber scaffold reinforced by the woven fabrics, both of which were composed of the same fiber material, polydioxanone (PDO) (Fig. 2), used for the endogenous tendon repair. Due to the simultaneous application of weaving and electrospinning technology, the strength of the scaffold was increased by at least 20 times up to 65 MPa, and the maximum suture strength was up to 167 N, which was similar to that of human rotator cuff tendon at about 170 N. Remarkably, the PDO-based composite also directed tendon cell behavior and desired nanofiber-mediated bioactivity *in vitro* and *in vivo*. The multi-layered scaffold implanted in an *in vivo* rat model was gradually more embedded in dense tissue, and the PDO fiber pattern was less visible by weeks 6 and 12. During the study period, the scaffold remained well integrated, and there was no stratification or separation of the layers (Fig. 3). This study designed a non-destructive technique that incorporated strong woven fabrics into the aligned electrospinning layers to achieve a strong but bioactive scaffold that could match the mechanical properties of tendons and guide the cell behavior. And the synthetic polymer that can well match the degradation time is also a crucial factor for the success of the scaffold.

**Figure 4.**
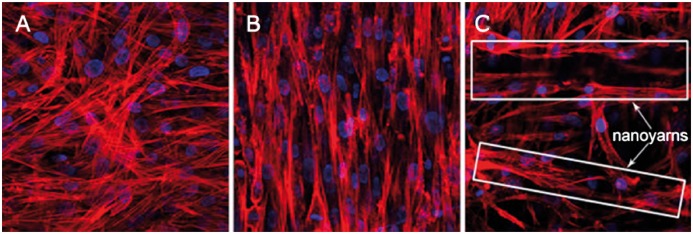
Confocal microscopy fluorescence images of the actin filaments (red) and nuclei (blue) of MSCs on the random nanofibrous scaffold (**A**), aligned nanofibous scaffold (**B**) and NRS (**C**) after 7-day culture. Magnifications of all images are 400×. (Adapted with permission from Ref. [58]. Copyright 2014 Elsevier Ltd)

As for the scaffold composed of both synthetic polymers and natural materials, Yang *et al.* [58] used electrospun nanoyarns as reinforcements to design an aligned nanoyarn-reinforced nanofiber scaffold (NRS) composed of poly(_L_-lactide-*co*-caprolactone) (P(LLA-CL)) and silk fibroin for tendon tissue regeneration. The composite with a highly oriented microstructure achieved high porosity of 79.2 ± 3.2% and large pore sizes of 551.41 ± 472.70 μm^2^. The tensile properties of the reinforced composite were also improved in the direction parallel to the aligned nanoyarns, which made the scaffold consistent with the mechanical requirements for tendon repair. The direction of the nanoyarns also had a good promoting and guiding effect on cell growth that bone marrow mesenchymal stem cells (MSC) cultured on the composite had a higher rate of proliferation and presented an aligned and prolonged growth pattern in the direction of the nanoyarns (Fig. 4). However, the distribution of cell in the NRS was not uniform, which might be caused by static culture. This study combines the advantages of synthetic P(LLA-CL) with excellent mechanical properties and biodegradability and natural biological silk fibroin with biocompatibility, hydrophilicity and low immunogenicity to obtain a balance between the porosity and mechanical properties of electrospun scaffolds. In the other study, Webb *et al.* [59] also investigated a poly(3-hydroxybutyrate-co-3-hydroxyhexanoate) (PHBHHx) scaffold with a PHBHHx fiber-reinforced collagen core used in a rat Achilles tendon repair model. The results showed that the reinforced PHBHHx scaffold had comparable breaking loads of 23.73 ± 1.08, which were significantly stronger than that of the native rat tendon of 17.35 ± 1.76 N. The reinforced PHBHHx scaffold with collagen core not only facilitated a regenerative cellular response but also subsequently remodeled the damaged tissue without causing an immune response or prolonging inflammation in the rat model over 40 days. Although the reinforced PHBHHx with a complex structure was different from the above regular reinforced scaffold, the addition of the fiber-reinforced collagen core favored cell proliferation and infiltration and facilitated the integration and remodeling of tendon tissue. The above two studies have demonstrated that synthetic polymers in conjunction with natural materials can be used as a scaffold material to offer a suitable structural morphology and mechanical environment that contribute to the successful application in ligament or tendon repair *in vivo*. However, due to differences in biochemical properties, how to make the two types of materials better integration of each other still need further study.

## Fiber-reinforced scaffolds for vascular tissue repair

Nowadays, there are about 17 million people died from the cardiovascular and cerebrovascular diseases in the world [60, 61], and the number is expected to reach 25 million by 2020. Autologous saphenous vein and mammary artery transplantation are the gold standards for clinical therapy, but the selection of vascular substitutes that can be used for these transplantation is very limited [62]. And there are still a series of clinical problems that cannot be solved, such as thrombosis, adverse immune response, neointimal hyperplasia, suture retention and poor mechanical properties, especially for small diameter vascular grafts [63]. Vascular tissue engineering has emerged as an effective way to generate a variety of vascular substitutes with potential functions [61, 64]. A wide range of natural and synthetic materials have been designed to simulate vascular ECM, but it is difficult for single materials to meet the requirements of the vascular scaffold due to the complexity of the structures and functions of the natural blood vessel.

**Figure 5.**
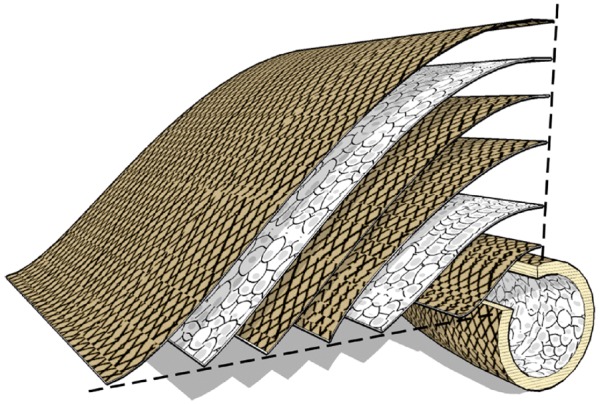
Multilamellar protein polymer microfiber composite vascular graft. The vessel wall has a multilamellar structure consisting of collagen microfiber (thickness 100 mm) embedded in protein polymer, and its orientation and density are established to achieve the mechanical design goals. (Adapted with permission from Ref. [67]. Copyright 2010 Elsevier Ltd)

Fiber-reinforced scaffolds have been the focus of vascular tissue research to prepare an ideal vascular scaffold with suitable structure, certain mechanical property, high cell compatibility and no immunogenicity. Pooyan *et al.* [65] reported a complete bio-based reinforced scaffold with a 3 D rigid percolating network, which had the similar profile features to natural ECMs in human blood vessels. The composite consisted of a cellulose acetate propionate matrix reinforced with cellulose nanowhiskers (CNWs), a kind of short fibers. The composite was confirmed by CNWs interconnected by strong hydrogen bonding to offer an improved mechanical system at body temperature. The ultimate tensile strength was increased from about 3.0 MPa to about 8.8 MPa. In this study, the good mechanical stability of the scaffold was due to the good dispersion and interconnection of the CNWs in the host matrix, forming a rigid percolating network. This cellulose-based reinforced scaffold in vascular tissue engineering utilizes its own chemical properties and the reasonable structural design to compensate for the lack of mechanical properties in order to give full play to controlled degradability, biocompatibility, reproducibility and environmental friendliness of cellulosic biomaterials. Likewise, Liu *et al.* [66] also designed a bilayer silk-based reinforced scaffold composed of an inner silk fiber-reinforced silk fibroin tube embedded in the outer nanofibrous silk layers as small diameter vessel prostheses. The outer layer of the grafts provided a suitable growth environment for fibroblasts and smooth muscle cells, while the inner silk fiber-reinforced films with heparin provided excellent blood compatibility for at least one month. The bilayer fiber-reinforced grafts not only provided better mechanical strength, burst pressure, and suture retention strength but also achieved a similar compliance to saphenous veins. Compared with the synthetics, natural biopolymers can better simulate the characteristics of ECM in human blood vessels while providing less thrombosis substitutes. However, the weak mechanical strength of these biopolymers limits their clinical applicability. The two examples indicate that the mechanical performance of natural polymeric materials reinforced by the fibers same material can be controlled and improved by changing the geometry and morphology of their component, the volume fraction of each phase and the distribution and relative positions of the phases, while not affecting its good biological properties. But the reinforcing unit is not limited to the same materials. For example, Caves *et al.* [67] designed a new type of cell-free arterial substitutes with a multilamellar structure by reinforcing a recombinant elastin-like protein with synthetic collagen microfibers for the regeneration of smaller diameter (<6 mm) grafts (Fig. 5). The breaking strength, compliance and suture retention strength of the reinforced scaffold were 239–2760 mm Hg, 2.8–8.4%/100 mm Hg and 35–192 gf, respectively, most of which were closely approximate to all target criteria. This study found that the mechanical properties of the composites could be adjusted by controlling the fiber orientation and volume fraction. Compared with the natural fibers, synthetic collagen fibers used as reinforcements also had a good performance in the design of the structural morphology, but their greatest advantage was to improve and balance the mechanical properties of vascular scaffolds.

**Figure 6.**
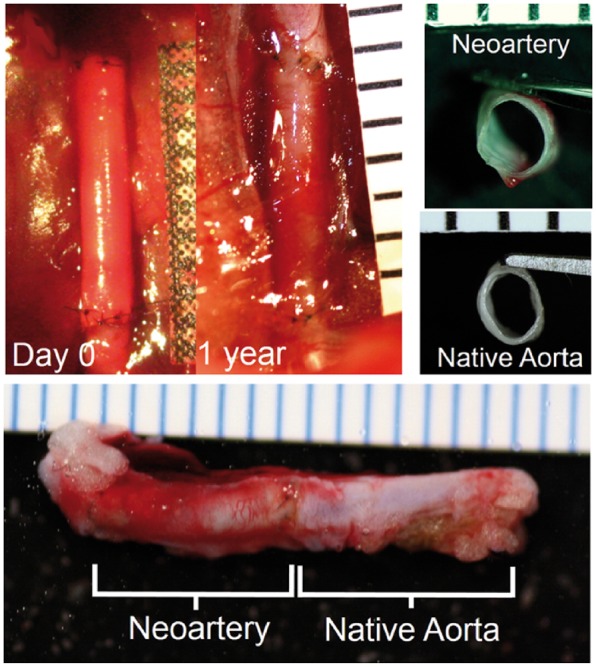
Gross morphology of neoarteries. Top left: transformation of graft into neoartery *in situ* over the course of 1 year. Top right: Transverse view of explanted neoarteries resembles that of native aortas. Bottom: Longitudinal view of explanted neoarteries resembles the adjacent native aorta. All ruler ticks are 1 mm. (Adapted with permission from Ref. [69]. Copyright 2013 Elsevier Ltd)

Some other studies prefer to take advantage of good mechanical properties of synthetic polymers to prepare vascular scaffolds that can remain the functional micro-environment under the complex loads. Liu *et al.* [68] designed a PVA-SbQ (Polyvinyl alcohol with styrylpyridinium pendent groups) fiber-reinforced PVA/Gelatin cryogel blood vessel, composed of an inner layer of PVA-SbQ fibers embedded in the cryogel. PVA-SbQ fibers provided good mechanical compliance in pulsating flow with an average pressure range of 40 mmHg to 140 mmHg for pulsating pressure signals. The compliance curve of the fiber-reinforced structure was similar to that of the human superficial femoral artery found in the literature. Among the mechanical properties, compliance under physiological stress is associated with intimal hyperplasia, which affects vascular patency. Thus, fiber-hydrogel vascular substitutes represent the promising to duplicate the compliance properties of natural blood vessels. In addition to achieving good results in improving structural and mechanical properties, these synthetic scaffolds have also made encouraging achievements in supporting cell growth and matrix formation. Allen *et al.* [69] prepared a small, resorbable arterial graft consisting of a rapidly degraded microporous poly (glycerol sebacate) (PGS) tube embedded in the reinforcing PCL fibers on the outer surface. The vast majority of grafts was completely absorbed after 30 days of implantation, except for rare PCL residues [70]. The graft was reconstructed to regenerate new arteries, which had an overall appearance similar to native rat aortas after one year of implantation in rat abdominal aortas (Fig. 6). In addition, the reinforced scaffold enhanced the regeneration of the nerves in the perivascular tissue, and the new arteries responded to vasodilators, which can regulate vasoconstriction, although the magnitude was different from native aortas. This study showed that cell-free scaffolds with rapid resorbability and elasticity could reconstruct damaged tissue and result in regenerative arteries with long-term stability. In general, these current findings confirm that the fiber-reinforced scaffold from natural or synthetic substances has the potential as vascular grafts with a range of diameter, and the follow-on *in vivo* studies still need to be conducted to provide the scaffold with porous structure, appropriate biocompatibility, hemocompatibility, mechanical properties and degradation behavior.

## Fiber-reinforced scaffolds for skin tissue repair

The skin, as the largest organ of the body, primarily serves as a barrier to protect wounds from infection and dehydration and controls the inward and outward passage of various substances. The large area skin defects will seriously affect physical health, and appearance and even lead to death. Although autologous transplantation and allograft skin grafting are the most commonly used method of repairing irreversible damage skin, their limitations, such as hyperplasia, infection and scarring, give rise to find a new alternative substitute for damaged skin tissue [71–73]. Skin tissue engineering has made some progress in the field of wound healing, especially in the case of burns without the availability of autologous skin. A series of skin scaffolds have been developed to achieve interconnected porous structure, good biocompatibility and biodegradation in skin tissue regeneration. However, recent studies found that scaffolds for skin or even load-bearing soft tissue have frequent defects that cannot meet stringent mechanical requirements due to their low stiffness but high tensile strength [74–76]. For a qualified skin scaffold, it should have the abilities to resist suture tension during implantation and maintain the function and structure after implantation [77, 78]. In the past years, fiber-reinforced scaffolds have used for the skin tissue and made a certain research progress. For example, Han *et al.* [79] designed a degummed silk fiber-reinforced silk nanofibrous scaffold, which had a good clinical application in soft tissue regeneration, especially for skin tissue repair and regeneration. The silk nanofibrous scaffolds possessed better suture retention strength, elongation at break and tensile strength without sacrificing morphology, porous structure and cytocompatibility. The suture retention strengths, elongations at break and tensile strengths were 3.2 ± 0.5 N, 20.89–20.92% and 1.70–1.74 MPa in the wet state. The moduli of the composite were 0.60 MPa in the wet state, similar to that of the skin rang from 0.13–0.66 MPa *in vivo.* The reinforced scaffolds also maintained excellent biocompatibility of silk fibers. The amniotic fluid-derived stem cells grew on the surface of the porous wall and formed a continuous monolayer after 12 days. In fact, unlike other soft tissue engineering, a variety of attempts are mainly focused on the natural materials contained in the skin ECM, such as collagens, fibrin and hyaluronic acid, for these materials is conductive to create a bioactive environment for supporting keratinocytes. Stark *et al.* [80] also reinforced collagen hydrogels by the modified hyaluronic acid fibers (Hyalograft-3 D) and then implanted the skin fibroblasts to present a genuine dermis-type scaffold. The reinforced skin scaffold had a superior epidermal architecture, including ultrastructure and regular differentiation, and the *in vitro* model of keratinocytes exhibited improved stability, which provided the basis for skin regeneration and homeostasis. The addition of fibers can use to improve and adjust the mechanical properties of the skin scaffold, while remaining their biocompatibility. The fiber-reinforced scaffold can be served as matrices for repair skin tissue and provide a new prospect for achieving scaffolds with appropriate mechanical properties.

## Fiber-reinforced scaffolds for the intervertebral disc repair

The intervertebral disc (IVD) consists of two distinct regions, the inner nucleus pulposus (NP) and the outer annulus fibrosus (AF). The primary function of IVD is to separate spinal vertebrae of the body and protect the spine from impact loads [81]. Four-fifths of the adults suffer from back pain with age, which is directly linked to intervertebral disc degeneration [82]. Up to now, invasive surgery, such as spinal fusion, intervertebral disc resection or artificial lumbar disc replacement, is the most common and effective treatment for patients with severe spinal disease [83, 84]. But the current treatments often result in other complications, such as accelerated degeneration of adjacent vertebrae. Tissue-engineered therapy has attracted more and more interest as a solution for traditional strategies, in particular the use of scaffolds with structures similar to IVDs, which is expected to regenerate and remodel the IVD tissues.

**Figure 7.**
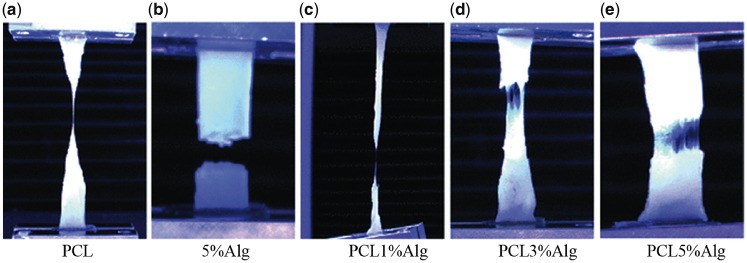
Images of single phase materials and composites undergoing tensile testing, at the point of failure. (Adapted with permission from Ref. [81]. Copyright 2014 Springer)

The IVD with a structure of proteoglycan hydrogels encapsulated into collagen fibers is much like fiber-reinforced composites. Thus, the fiber-reinforced scaffolds not only have more viable structures and IVD-like components, but also provide sufficient biomechanical properties to simulate the characteristics of the two regions, NP and AF. As described by Ambrosio *et al.* [85], a PET continuous fiber reinforced poly(2-hydroxy-ethyl methacrylate) (PHEMA)-PCL hydrogel with semi-interpenetrating networks (s-IPNs) has the potential to be used as an artificial disc due to their high biocompatibility, permeability and hydrophilicity. When the PHEMA-PCL hydrogel was reinforced by about 40 vol% PET fibers with a winding angle from 45° to 65°, the maximum stress and modulus increased from 12 to 17 MPa and from 30 to 73 MPa, respectively, which were typical values of the canine lumbar intervertebral disc. When the PET fiber volume increased to 50%, the maximum stress and modulus increased up to 20 MPa and 129 MPa, respectively. Furthermore, this study indicates that the hydrophilicity and mechanical properties of the IVD scaffold can be controlled, and the PET reinforced PHEMA-PCL may allow the native tissue to grow inwardly when the PCL degrades and then leaves the void in the network. As opposed to using synthetic polymers as the matrix material, some other studies also utilize fibers as reinforcements to optimize the mechanical behavior of the natural polymer hydrogel for IVD tissue engineering. This may be due to the fact that the fiber-reinforced natural hydrogel has the composition and structure more similar to the natural IVD, which is conducive to imitate the anisotropic mechanical properties. Strange *et al.* [81] provided a methodology to design the tensile and compressive mechanical properties of the fiber reinforced composites to create functional engineered IVD scaffolds more closely similar to the complex structure of native IVD and other tissues, through modulating the allocation of the fiber and matrix phases. The composite was composed of thick 3D electrospun PCL fibers infiltrated with alginate (Alg) hydrogels. The tensile and compressive properties of PCL-Alg composites were dominated by the presence of PCL fibers and the modulus of hydrogel phase, respectively (Fig. 7). The PCL fibers improved the tensile stiffness and strength of the composite and mitigated the compression time-dependent response, while the Alg hydrogel supported and distributed compressive loads, through fluid pressurization. Likewise, Thorvaldsson *et al.* [86] also designed a PCL nanofiber-reinforced gellan gum gel scaffold for the NP regeneration by using electrospinning and air brush spraying. The reinforced composite with a small amount of nanofibers had enhanced mechanical properties and rheological properties similar to those of human NP, while large amounts of nanofibers resulted in a decrease in the shear modulus of the gel structure. And the modulus of the composite was within the range of that of native human NP. The IVD is the main mechanics and movement unit in the spine, which needs to bear a variety of loads such as compression, shear and torsion. The natural fiber-reinforced scaffold may not be able to reach the critical mechanical requirements of the IVD scaffold, therefore most of the research use synthetic polymer fibers as a reinforcing material in IVD tissue engineeting. The fiber-reinforced scaffold provides the potential to be an ideal IVD scaffold, which can simulate the complex internal and external structure of the natural IVD and have sufficient biomechanical properties to bear and transmit the load, as well as limit the excessive movement of the spine.

## Fiber-reinforced scaffolds for corneal tissue repair

There are about 10 million people around the world suffering from blindness due to corneal diseases every year [87]. At present, human corneal transplantation is the only feasible and effective treatment for corneal blindness, however, the donor cornea with a high quality cannot satisfy the needs of patients, especially with the growth of the elderly population. In the past few years, tissue-engineered corneas have made some progress in preparing substitutes with similar microstructure and properties of native corneal tissue. The success of a tissue-engineered cornea is significantly dependent on the scaffold’s performance similar to the microstructure and properties of the corneal ECM, whose major components are collagen fibrils and proteoglycans. The use of fiber reinforcements allows the biomaterials to have biological properties similar to those of the cornea, such as sufficient mechanical properties, morphological integrity and transparency of corneal tissue repair [88–90]. For example, Long *et al.* [91] designed a series of silk fibroin reinforced collagen-based membranes as corneal implants to achieve suitable mechanical properties. The water content of the composite membrane was determined by the content of silk fibroin. The collagen-based membrane with 10 wt % silk fibroin (CS10) possessed the optimal mechanical properties. In particular, CS10 had a high suture retention strength, which could be sutured in the rabbit eyes as a whole. The membrane also possessed better optical properties and higher light transmittance due to its highly organized construction and suitable components. When the CS10 membrane was used for lamellar keratoplasty, the epithelium was completely formed within 35 ± 5 days and the transparency was rapidly restored within the first month. In the other study, Tonsomboon *et al.* [92] developed a transparent gelatin nanofiber-reinforced alginate hydrogel to simulate the microstructure of the cornea. Both gelatin and alginate are inexpensive, non-immunogenic and readily available natural polymers. The elastic modulus of the composite at 0.45–0.5 MPa was very close to that of natural cornea at 4.9–0.579 MPa [24, 93, 94]. In addition, optical transparency was also desirable, because the diameter of the uniformed gelatin fibers was relatively small at 67 ± 7 nm when compared with the visible wavelength at 400–700 nm. The study provides a new prospect for the commercial manufacture of improved transparent hydrogel scaffolds as a cornea substitute by using two inexpensive natural polymers: gelatin and alginate. In general, it has to be emphasized that the incorporation of fibers in the host matrices can be a meaningful strategy to improve the water content, mechanical integrity and transparency of the cornea scaffold. The fiber-reinforced composite may provide a promising approach for designing the corneal scaffolds that can be used for human corneal transplantation.

## Conclusion

As the review has attempted to describe in conjunction with current research, fiber-reinforced scaffolds provide an effective platform for engineer a variety of ideal scaffolds for repairing and regenerating defective or damaged soft tissues and organs. Fibers with extremely high aspect ratios are particularly suitable to be used as a controllable element for the manufacture of biomaterials with various morphology and sufficient mechanical properties. The reinforced scaffolds are typically made of the gel-based systems reinforced with various fibers, including natural and synthetic materials, as summarized in Table 1. The main advantage of the reinforced composites is that they can capture the benefits of each material to overcome the limitations of each single-component scaffold. In addition, their another non-negligible advantages are that they can better mimic the microstructure and composition of natural soft tissues that are composed of collagen fibers embedded in a hydrogel-like matrix of elastin and meet the long-term functional requirements of biomechanics. Over the past few years, the fiber-reinforced scaffold with their unique properties and functions play a key role in improving the biomechanics, biocompatibility, bioactivity, integration and degradation of artificial composite scaffolds in soft tissue repair and regeneration. In fact, fiber-based biomaterials with a wide range of morphological and mechanical properties have been widely used as reinforcing additives in soft tissue engineering to produce the desirable scaffold similar to ECM components in animal models and in clinical applications. However, a series of new barriers in the research are emerged that researchers continue to overcome before successfully utilizing them in the *in vitro* research and clinical applications.

**Table 1.** Examples of fiber-reinforced scaffolds in soft tissue engineering mentioned in the article

Fibers	Matrix	Applications	Limitations	Advantages
PGA	PLGA	Cartilage repair	Low shear property	Controlled porosity with interconnected pores, controlled pore structure, biodegradable,
PCL	GelMA hydrogel	Cartilage repair	Faster relaxation, lower equilibrium modulus	Bioresorbable, biodegradable, high stiffer, efficient cell-seeding properties, controlled pore structure,
	PGS	Aortas repair	Less uniform distribution, some residual material, substantial inflammation	
	Alginate hydrogel	NP repair	No tensile-compressive nonlinearity, low structural resilience	
	Gellan gum gel	IVD repair	Insufficient porosity, inadequate interconnectivity	
(pHMGCL)/PCL	GelMA hydrogel	Cartilage repair	Insufficient integration, low structural resilience	Hydrophilic, high degradation rate, low glass transition temperature, cytotoxicity
Silk	Silk hydrogel	Cartilage repair	Insufficient stiffness, insufficient Compressive strength, insufficient tensile strength	Superior biocompatibility, bioactive, degradability, hydrophilicity, non-toxic, non-immunogenic,vascular support,
		Vascular repair		
		Skin repair		
	Collagen hydrogel	Corneal repair		
	Chitosan/glycerophosphate hydrogel	Cartilage repair		
Collagen	Collagen–chondroitin- 6-sulfate	Ligament and tendon repair	Low tensile strength	Highly bioactive, Bioresorbable, non-immunogenic,
PET	HydroThaneTM	Ligament and tendon repair	Unsatisfactory porosity	Cellular compatibility, hydrophilicity, biodegradable, biocompatible, high permeability
	PHEMA-PCL hydrogel	IVD repair	Low water content	
PDO	PDO	Endogenous tendon repair	Uncontrollable porosity	Bioactivity, design flexibility, biodegradable, biocompatible
PHBHHx	Collagen hydrogel	Tendon repair	Insufficient elasticity, insufficient strength	Design flexibility, cellular compatibility, non-immunogenic, delayed biodegradability
CNWs	Cellulose acetate propionate	Vascular repair	Insufficient porosity, insufficient pore size	Cellular compatibility, mechanical stability, controlled degradability, biocompatibility, reproducibility
Hyaluronic acid fibers	Collagen hydrogel	Skin repair	Unsatisfactory homeostasis, incomplete differentiation	Biodegradable, biocompatible, nonimmunogenic
Gelatin	Alginate	Corneal repair	Low transverse stiff	Hydrophilic, transparent, non-immunogenic, biocompatibility

Despite the remarkable achievements of current research, there is still a need to develop advanced theories and satisfactory intensive processing techniques to achieve the fiber-reinforced scaffolds with favorable morphological structure, optical properties, electrical properties, biocompatibility, biochemical and biological properties, especially adequate mechanical properties. Aiming at these goals, there are some important issues that we should focus on in the future. Firstly, With the development of materials engineering, life sciences and medicine, the next generation scaffold will be made of more intelligent and diverse materials, with a more satisfactory structure and function. Thus, an in-depth systematic study should be conducted on all the impact factors that affect the performance of the reinforced scaffold, such as the volume fraction, aspect ratio, direction and arrangement of the fiber. Secondly, the design of new composites depends to a large extent on its manufacturing technology and experimental conditions, which we should be concerned about. Finally, the fiber-reinforced composites seeded with active enzymes, cells and antibodies are still a further study direction, which may make a difference on optimizing the next generation of engineered scaffolds for soft tissue repair.
